# Hydrogel-Based Localized Nonviral Gene Delivery in Regenerative Medicine Approaches—An Overview

**DOI:** 10.3390/pharmaceutics12080752

**Published:** 2020-08-10

**Authors:** Natalia Carballo-Pedrares, Isaac Fuentes-Boquete, Silvia Díaz-Prado, Ana Rey-Rico

**Affiliations:** 1 Cell Therapy and Regenerative Medicine Unit, Centro de Investigacións Científicas Avanzadas (CICA), Universidade da Coruña, 15071 A Coruña, Spain; natalia.carballo@udc.es (N.C.-P.); i.fuentes@udc.es (I.F.-B.); s.diaz1@udc.es (S.D.-P.); 2 Departamento de Fisioterapia, Medicina y Ciencias Biomédicas, Facultad de Ciencias de la Salud, Universidade da Coruña (UDC), Instituto de Investigación Biomédica de A Coruña (INIBIC), Complexo Hospitalario Universitario de A Coruña (CHUAC), Servizo Galego de Saúde (SERGAS), 15071 A Coruña, Galicia, Spain

**Keywords:** musculoskeletal tissue, cardiovascular tissue, wound healing, nervous tissue, gene therapy, controlled delivery, hydrogels, nonviral vectors

## Abstract

Hydrogel-based nonviral gene delivery constitutes a powerful strategy in various regenerative medicine scenarios, as those concerning the treatment of musculoskeletal, cardiovascular, or neural tissues disorders as well as wound healing. By a minimally invasive administration, these systems can provide a spatially and temporarily defined supply of specific gene sequences into the target tissue cells that are overexpressing or silencing the original gene, which can promote natural repairing mechanisms to achieve the desired effect. In the present work, we provide an overview of the most avant-garde approaches using various hydrogels systems for controlled delivery of therapeutic nucleic acid molecules in different regenerative medicine approaches.

## 1. Introduction

Recent progress of our understanding of how cells utilize nucleic acids (NA) has focused the attention to develop a range of original (plasmid DNA (pDNA)) and emerging (RNA interference (RNAi) and messenger RNA (mRNA)) nucleic acid candidates for treatment of a wide range of diseases [1,2], to trigger (pDNA, mRNA) or suppress (small interfering RNA (siRNA) and micro RNA (miRNA)) the expression of specific genes and transcription factors [2,3].

Protein expression via pDNA and mRNA involves the internalization of these molecules into the cell’s nucleus and cytoplasm, respectively [4]. In addition, RNAi has emerged as a gene regulatory mechanism of silencing gene expression based on the blockage, degradation, or both of specific mRNA [5]. Particularly, siRNAs are 19–27 nucleotides long double-stranded RNA molecules having an important role in degrading mRNA of disease-related genes [6]. Moreover, miRNAs are endogenous single-stranded 19–25 nucleotide long RNA molecules and mid-matched based pairing that play a pivotal role in endogenous gene regulation [7]. Beyond to mediate gene silencing similarly to siRNA, miRNA can regulate gene expression directly and regulating the expression of other mRNAs [4].

Although the delivery of naked NA molecules into the cells is considered the safest way of transfection, this process is highly ineffective due to the electrostatic repulsions occurring at physiological pH between the anionic NA molecules and the negatively charged plasma membrane [8]. Therefore, the internalization of these NA into the cells is normally mediated by gene carriers or vectors in order to achieve an effective gene transfer. These vectors can be categorized as viral or nonviral based on the nature of the carrier involved. Viral vectors rely on the natural cellular entry pathways of viruses from which they are derived, being highly efficient at internalizing these NA molecules into the cells [9]. However, gene therapy via viral vectors carries important shortcomings due to their risk of insertional mutagenesis, inherent cytotoxicity and/or immunogenicity [2], and tumorigenic risk [10,11]. Herein, gene delivery via nonviral systems is nowadays at the forefront of gene therapy [12]. Nonviral systems involve the complexation of NA molecules with positively charged gene carriers like polycations (polyplexes), cationic or ionizable lipids, and lipid-like molecules (lipoplexes) to promote their uptake in the cells [3]. Despite their biosafety as compared with viral counterparts, gene transfer via nonviral systems is precluded by some obstacles associated to the vector itself (fast degradation, short half-life, serum neutralization, instability in physiological fluids, and aggregation tendency) as well as its internalization and cell trafficking mechanisms of the NA molecule to initiate the expression of the transgene [3,13,14,15]. Unlike to pDNA, mRNA does not need to enter in the cell nucleus to be functional, requiring only the translational machinery in the cytosol for expression of its protein product [16]. Therefore, a superior gene transfer efficiency has been reported for mRNA molecules compared with pDNA [17]. However, a higher instability and host immunogenicity have been reported for mRNA molecules [18]. Thus, is critical to understand the properties and functions of different NA molecules to select the appropriate carrier for effective transfection.

The design of nonviral gene delivery systems may help to overcome these issues by maintaining elevated concentrations of foreign sequences in the cellular microenvironment while protecting them against degradation and/or reducing their immunogenicity in order to achieve selective and durable transgene expression into the specific target sites [13]. Specifically, tissue regeneration can be improved by modulating the extent and the distribution of transgene expression within and around the injury [19].

### 1.1. Requirements for Localized Gene Delivery in Tissue Regeneration

Tissue regeneration following disease or injury requires exogenous signals to enhance the natural healing processes and suppress inhibitory pathways [15]. Owing to the essential role that the extracellular matrix (ECM) plays in maintaining the physiological stability of the microenvironment and guiding tissue-specific function, biomaterials have been engineered in an effort to support tissue regeneration and serve as vehicles for cell transplantation, promoting survival, differentiation, and engraftment [15]. Likewise, these biomaterial scaffolds may be exploited to deliver therapeutic gene molecules, providing a controlled release of these agents in desired locations as a means to avoid clearance mechanisms and reinforce their stability in the physiological milieu [2]. The use of “gene medicines” offers an alternative to small drugs and recombinant growth factors that are prone to nonspecific effects on various cellular systems and may induce resistance once the innate physiological mechanisms are induced by the cells to overcome the drug effects [2]. Further, compared with recombinant growth factors that exhibit half-life in the range of minutes and a rapid inactivation in physiological conditions, gene therapy offers the possibility of directly transferring genes encoding for the therapeutic factor into the target cell population [20]. Therefore, by transfecting specific gene sequences into cells, overexpressing, or silencing the original gene, their biological functions can be regulated to achieve the desired effect [21].

### 1.2. Hydrogels as Vehicles for Nonviral Gene Delivery

Hydrogels constitute a class of biomaterials formed by self-assembling or crosslinking of water-soluble polymers into a network [22]. The porous and hydratable structure of hydrogels induces their gelation and swelling in the biological microenvironment, enabling their local administration by injection without invasive surgery [22]. Hydrogels can be fabricated via physical (such as hydrogen bonding and ionic and hydrophobic interactions) or chemical (such as photopolymerization or Michael-type addition reaction) crosslinking mechanisms [23]. Moreover, hydrogels can be engineered to exhibit adapted properties to the tissue to be repaired as 3D-bioprinted constructs [24,25] and/or injectable [26,27], stimuli-responsive [28,29], or adhesive systems [30,31].

Due to their capability to mimic the properties of the ECM, hydrogels can improve the survival, differentiation, and integration of host cells [32]. Likewise, in an effort to control the release kinetics and preserve the activity of therapeutic biomolecules, hydrogels have been widely investigated as gene delivery systems [15]. Various hydrogels systems based on natural polymers such as alginate [33,34,35,36,37,38,39]; cellulose [40]; chitosan [41,42,43,44] (Table 1); collagen [45,46,47]; dextran [48]; fibrin [17,49,50,51,52,53,54,55]; pullulan [56] (Table 1); gelatin [57,58,59,60]; hyaluronic acid (HA) [61,62,63,64,65,66,67,68,69] (Table 1); or synthetic ones as polyethylene-glycol (PEG) [70,71,72,73,74,75,76,77,78,79,80,81,82,83,84,85] (Table 2), poly(N-isopropylacrylamide) (PNIPAm) [86], polyurethane [87], or poly(organophosphazene) [88] (Table 2) have been studied as delivery systems of therapeutic NA molecules in various tissue engineering approaches.

Encapsulation of pDNA complexes into biocompatible hydrogels has showed to be a powerful approach to achieve a localized delivery into the target cells, protecting the therapeutic gene against degradation and enhancing the transgene expression into the target cell populations [33,34,35,36,37,38,39,40,41,42,45,50,51,52,53,54,55,57,61,64,65,66,67,68,69,70,72,73,74,77,83,87,88,89,90,91].

Similarly, controlled delivery of RNA molecules from hydrogels networks may enhance local and sustained siRNA [43,44,48,49,56,59,71,75,76,78,79,85,86] and miRNA [46,47,60,62,63,80,81,82,84,92] delivery limiting undesired targets [14], and protect mRNA nanoparticles from the biological environment improving their cellular access [17,58].

The aim of this review is to provide an updated overview from the state-of-the-art on the use of hydrogels as controlled gene delivery systems in regenerative medicine approaches with a special focus on musculoskeletal tissue repair, cardiovascular tissue repair, wound healing, and neural tissue repair. To this end, the main hydrogel systems used for controlled delivery of pDNA, mRNA, siRNA, and miRNA molecules are discussed.

## 2. Hydrogel-Mediated Gene Delivery in Regenerative Medicine

### 2.1. Musculoskeletal Tissue Repair

**Figure 1:**
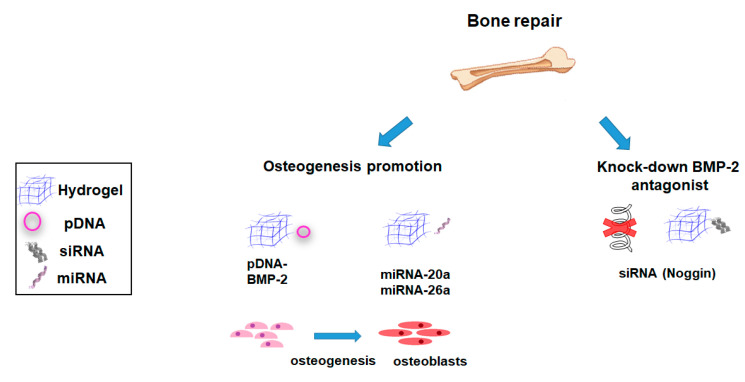
Main strategies involving the use of hydrogel-based nonviral gene delivery systems for bone tissue repair.

**Figure 2:**
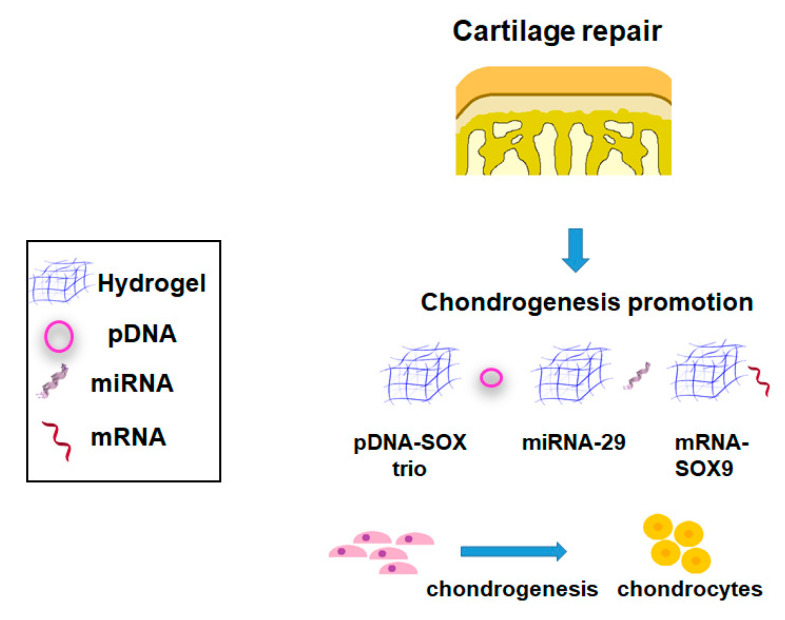
Main strategies involving the use of hydrogel-based nonviral gene delivery systems for cartilage tissue repair.

Musculoskeletal tissues are diverse and significantly differ in their ability to repair spontaneously upon injury [93,94]. Herein, while articular cartilage has a very limited ability to self-repair, most fractures of long bones, differently to large segmental defects, heal by themselves. Further, a poor quality tissue is currently associated to tendons self-repairing process [95]. The first approaches to repair these tissues included delivering of instructive or inductive proteins like growth factors to promote natural tissue regeneration. However, providing sufficient local concentrations of these proteins necessary for tissue regeneration can lead to severe side effects. In contrast, gene therapy can yield sustained local production and secretion of proteins in sites of injury by directly transferring genes encoding for these therapeutic factors [13]. Combination of tissue engineering strategies with adapted gene transfer vectors represent a promising alternative for improved tissue regeneration. Significant research has been developed using different hydrogel systems to deliver various therapeutic NA molecules, mostly focused on bone and cartilage reparative approaches (Figure 1 and Figure 2; Table 1 and Table 2).

#### 2.1.1. Bone Tissue

The use of gene-activated matrices (GAMs) has emerged as a potential approach to promote bone regeneration [96]. In its original description, the GAM consisted of a collagen matrix loaded with pDNA encoding for bone morphogenetic protein 2 (BMP-2) to promote bone formation in vivo. This concept has been now extended to combine hydrogel-based biomaterials and vectors. This allows to provide a localized delivery of the therapeutic transgene at the place of the lesion while providing a microenvironment that mimics the ECM from the native tissue [96]. BMP-2 has for decades been the gold standard osteogenic factor for bone regeneration. Herein, nonviral delivery of pDNA encoding for BMP-2 has been extensively studied in bone regeneration approaches [33,34,35,36,37,38,41,42,70] (Figure 1; Table 1 and Table 2).

Wegman et al. investigated the efficiency of bone formation by using an alginate-based hydrogel loaded with pDNA encoding for BMP-2 (pDNA-BMP-2) [35]. This nonviral GAM was combined with goat multipotent stromal cells (gMSCs) and ceramic granules and implanted intramuscularly in goats. Transfection of cells with this DNA delivery system led to stable expression of BMP-2 for 16 weeks, promoting osteogenic differentiation and subsequent bone formation. A similar trend was observed by delivering pDNA-BMP-2 via gelatin-based hydrogels in a calvarial bone defect from mice [90].

An analogous strategy was involved to promote alveolar bone regeneration for the treatment of periodontal diseases. To this end, an injectable chitosan-based hydrogel scaffold containing pDNA-BMP-2-loaded chitosan nanoparticles was developed. The system showed excellent cytocompatibility and led to enhanced endogenous repair of alveolar bone [41,42].

In another approach to promote osteogenesis, a nano-type hydrogel (nanogel) composed by carboxymethylcellulose (CMC) complexed with branched cationic poly(ethyleneimine) (PEI) was synthetized. When this nanogel was loaded with pDNA encoding for the transcription factor osterix (OSX), a successful osteogenic differentiation of MSCs was notified [40].

Inhibitory molecules and antagonists responsible for maintaining tissue homeostasis can preclude tissue healing when employing regenerative medicine strategies [49]. In this scenario, the use of siRNA against these molecules has emerged as a potential tool to modulate the expression of these markers augmenting tissue healing [14] (Figure 1; Table 1 and Table 2). Owing to its role on BMP regulation being upregulated in response to high BMP-2 concentrations, Noggin has been selected as a target for many gene delivery researches to promote bone regeneration [44,49,71,78]. The use of siRNA against noggin constitutes a potential approach to this end, as it can knock-down this BMP antagonist in a temporary manner [44]. Efficient noggin suppression has been reported by delivery of siRNA from chitosan hydrogels leading to an increased bone healing in a mouse calvarial defect model [44]. Similar results were obtained using poly-d,l-lactic acid-p-dioxanone/polyethylene glycol block co-polymer (PLA-DX-PEG) hydrogels loaded with siRNA against noggin. The proposed system induced ectopic bone formation in mice, without significant adverse effects [71].

Concomitant delivery of siRNA and miRNA has showed to be a powerful tool to promote bone regeneration as it can regulate gene expression at the transcriptional or post-transcriptional levels [81]. Nguyen et al. reported that localized and sustained presentation of siRNA against noggin (siNoggin) and miRNA-20a (inhibitor of peroxisome proliferator-activated receptor gamma; PPAR-γ) from in situ forming poly(ethylene glycol) (PEG) hydrogels, enhances osteogenic differentiation of encapsulated human bone marrow-derived MSCs (hMSCs) [55]. Further, when implanted in a calvarial bone defect model, hydrogels containing encapsulated hMSCs and miRNA-20a resulted in more bone formation compared with those defects treated with hydrogels containing hMSCs without siRNA or with negative control siRNA [81]. In an innovative approach, Huynh et al. achieved a light-triggered RNA release profile via photodegradable, dual-crosslinked hydrogels [78]. Hydrogels loaded with PEI complexes of siNoggin and miRNA-20a led to increased hMSCs osteogenesis in vitro. Of note, RNA release from these photodegradable hydrogels could be accelerated upon UV application [78].

An avant-garde approach to improve the osteogenesis of encapsulated MSCs is based on the delivery mechanosensitive miRNAs biomolecules that can drive MSCs fate in injectable hydrogels [97]. Thereby, several mechanosensitive miRNAs have been identified and their efficiency to promote MSC osteogenesis have been described [97] (Figure 1; Table 1 and Table 2). Carthew et al., designed a gelatin–PEG hydrogel for in situ transfection of MSCs via miR-100-5p and miR-143-3p PEI complexes [82]. In situ transfection of MSCs promoted a higher osteogenic differentiation compared with encapsulation of previously transfected MSCs [82]. Li et al. identified a miRNA (miR-26a) that positively regulates angiogenesis–osteogenesis coupling [80]. When loaded in a heparin/hyaluronan/PEG scaffold and implanted in a critical-size calvarial bone defect, miR-26a optimized bone regeneration by simultaneous regulation of endogenous angiogenesis and osteogenesis processes [80].

In order to achieve a sustained and localized delivery of siRNA while preventing its degradation, a hybrid nanoparticle (NP)/hydrogel system was developed [79]. This system comprised siRNA against the negative regulator of bone formation WW domain containing E3 ubiquitin protein ligase 1 (Wwp1) complexed to NPs and subsequently entrapped within PEG-based hydrogels. Knockdown of Wwp1 using siRNA/NPs hydrogels showed significantly increased bone formation and accelerated healing in a murine mid-diaphyseal femur fracture [79].

#### 2.1.2. Cartilage

Despite their great potential, the use of hydrogels as controlled gene delivery systems for cartilage repair is still a developing strategy [20] (Figure 2; Table 1 and Table 2). Hereof, both chondral and osteochondral units are specially promising for polymeric gene delivery due to the limited blood flow to the region that could impair DNA delivery [83]. In an interesting adaptation, Gonzalez-Fernandez et al. designed three-dimensional (3D) printed pore-forming bioinks that provide a spatio-temporally defined gene delivery by modulating its porosity [38]. To this end, they involved alginate-methylcellulose hydrogels loaded with plasmids encoding for either osteogenic (BMP-2) or chondrogenic (transforming growth factor beta 3 (TGF-β3), BMP-2, and the sex-determining region Y box 9 (SOX9)) genes to produce mechanically reinforced, gene-activated scaffolds. Resulting delivery systems promoted osteogenesis and chondrogenesis of MSCs respectively, in vitro. When implanted in vivo, these bioprinted constructs supported the development of a vascularized, bony tissue overlanded by a layer of stable cartilage. A similar tendency was observed by implantation of a oligo [poly(ethyleneglycol) fumarate] (OPF) hydrogel bilayered scaffold simultaneously loaded with DNA encoding for runt-related transcription factor 2 (RUNX2) and SOX5, SOX6, and SOX9 (SOX trio) in a rat osteochondral defect model [83] (Figure 2).

Yang et al. synthetized a hybrid hydrogel composed of poly(N-isopropylacrylamide) (pNIPAAm) and layered double hydroxides (LDHs) for siRNA against glyceraldehyde-3-phosphate dehydrogenase delivery in osteoarthritic chondrocytes cultures [86]. Results showed a significant reduction (82–98%) of gene expression after 6 days of culture.

In contrast to siRNA being focused on downregulation of intracellular mechanisms, hydrogel-mediated mRNA controlled delivery is primarily intended to promote tissue formation or certain aspects of cellular response, such as differentiation, reprogramming, and protein secretion [3]. By these lines, a GAM based on fibrin-based hydrogel activated with mRNAs encoding for transcription factors (TF) SOX9 (cartilage) and MYOD (muscle) and loaded with hMSCs was developed [17]. Results from this study showed a higher and faster TF expression in hMSCs when using mRNA-GAMs as compared with pDNA-GAMs, resulting in enhanced synthesis of cartilage and muscle-specific markers in vitro.

#### 2.1.3. Other Tissues

**Figure 3:**
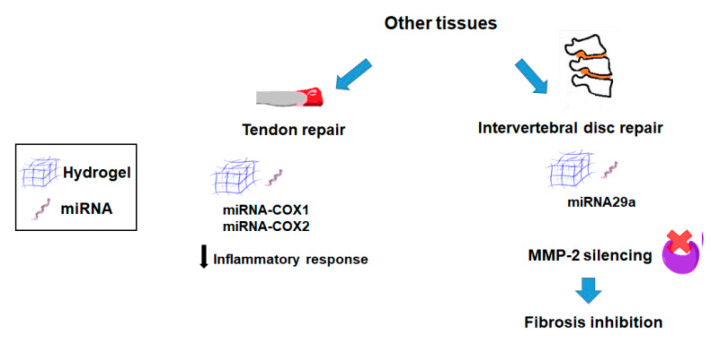
Main strategies involving the use of hydrogel-based nonviral gene delivery systems for repairing other musculoskeletal tissues.

To reduce flexor tendon adhesions, Zhou et al. developed a sustained gene delivery system composed of cyclooxygenase (COX-1 and COX-2)-engineered miRNA plasmid/nanoparticles embedded in a HA hydrogel [92]. This plasmid/nanoparticle hydrogel system significantly downregulated COX-1 and COX-2 expression in the tendon tissue and the surrounding subcutaneous tissue from a chicken model of tendon injury (Figure 3; Table 1).

In another attempt, Feng and co-workers designed an adapted RNA-based GAM to treat intervertebral disc degeneration (IDD). To this end, the authors combined a microRNA-29 (miR-29) exhibiting a potent fibrosis suppression capability, with matrix metalloproteinase (MMP)-degradable scaffold encapsulating MMP-responsive micelles due to the overexpression of these enzymes on IDD. For in situ formation of polyplex micelle-encapsulated hydrogels, these systems were comprised of cationic block copolymers designed to complex mir-29a mixed with PEG gelation precursors and MMP-cleavable peptide crosslinkers. These GAM resulted in an effective MMP-2 inhibition in IDD tissues inhibiting the fibrosis process and reversing IDD in a rabbit animal model [84] (Figure 3; Table 2).

### 2.2. Cardiovascular Tissue Repair

Cardiac tissue damage caused by myocardial infarction (MI) is one of the leading causes of death worldwide [98]. Most of the cardiovascular diseases are a part of a lifelong process of atherosclerosis, vascular inflammation, and its complications [99]. Common treatments to treat cardiovascular diseases include pharmacotherapy, left ventricular assist devices, heart transplantation, or cell-based cardiac therapy, but often failed to provide ideal regeneration for diseased cardiac tissue [100]. Additionally, stem cell therapy has demonstrated beneficial effects on cardiovascular therapy, although its use is still limited for several inconveniences limiting a successful cell-based therapy in MI [101,102]. The difficulty of pharmacologically targeting receptors and intracellular pathways involved in the pathogenesis of heart failure, had led researchers to propose gene therapy as alternative therapeutic approach [63,103,104,105].

**Figure 4:**
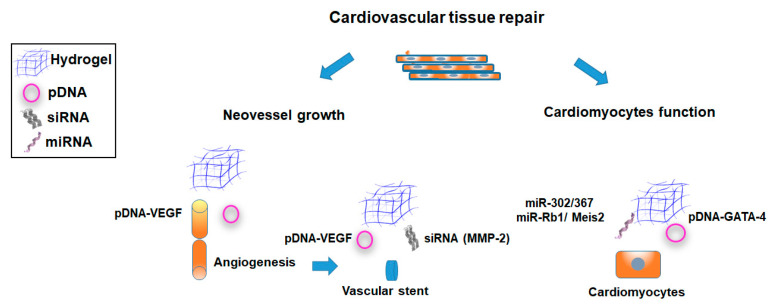
Main strategies involving the use of hydrogel-based nonviral gene delivery systems for cardiovascular tissue repair.

The progressive nature of the cardiac disease is mirrored in its manifestations. Exercise-induced ischemia, unstable angina, infarction, and ischemic heart failure are symptoms of different stages of the same disease process [106]. However, these events are usually considered separate therapeutic targets and different therapeutic genes are involved [99]. Herein, in the earlier stages of the cardiovascular disease the focus has been on the induction of neovessel growth [107], while in the heart failure setting the goal has been to improve cardiomyocytes function [108] (Figure 4; Table 1 and Table 2).

As the basic mechanisms of angiogenesis and blood vessel formation are already well-known, therapeutic vascular growth is a potential treatment option for ischemic myocardium. Among the growth factors used to this end, the members of the vascular endothelial growth factor (VEGF), fibroblast growth factor (FGF), and the hepatocyte growth factor (HGF) families constitute the most promising candidates (Figure 4). By these lines, pDNA has emerged as one of the preferred nonviral systems for the delivery of angiogenic factors to promote revascularization as they avoid some concerns (such as hemangioma formation) reported with the transference of same factors via viral vectors [10,11]. Controlled delivery of pVEGF complexes from various hydrogel systems has been reported to promote a localized neovascularization in different cardiovascular reparative approaches [39,50,52,57,61].

An important limitation of the incorporation of nonviral gene transfer vectors into hydrogels is the failure of loading high DNA concentrations because of their tendency to aggregate. To prevent pDNA polyplexes aggregation and inactivation, a Caged Nanoparticle Encapsulation (CnE) was involved [52,55,68]. Encapsulation of polyplexes containing pVEGF into MMP-degradable PEG hydrogels by CnE technology induced extensive angiogenesis in a choriallantoic membrane (CAM) in vitro assay. More recently, a nonviral gene delivery system using PEI-functionalized graphene oxide nanosheets (fGO) complexed with pVEGF was formulated and incorporated in methacrylated gelatin (GelMA) hydrogel to promote controlled and localized gene therapy [57]. When injected in a rat model with acute myocardial infarction, a significant increase in myocardial capillary density at the injected peri-infarct region and reduction in scar area were noted when compared with the controls.

Coronary stent implantation represents a common practice in interventional cardiology. However, restenosis remains by far the main complication of this technique. Stent coating with a polymeric layer that included a therapeutic gene has become in a potential therapy to overcome this issue [50,56,58]. In order to alleviate the risk of stent thrombosis and neointima formation, a fibrin nanobiohybrid hydrogel based on endovascular stent device was developed [50]. Hydrogel simultaneously delivered proangiogenic VEGF and angiopoietin-1 (Ang) genes loaded in nanoparticles. In vivo experiments in balloon injured canine femoral artery, demonstrated a significantly enhancement on re-endothelialization of injured artery attenuating stenosis and preventing neointima formation. In another approach, a cationized pullulan hydrogel was prepared to cover bare metal stents and act as delivery systems of siRNA or gene silencing of MMP-2 into rabbit arterial walls [56]. Results from this study showed a modulation of siRNA release by the presence of the cationic groups, compared with noncationized pullulan hydrogels. Additionally, when implanted in rabbit balloon-injured carotid arteries, these systems induced an uptake of siRNA into the arterial wall and a decrease of pro-MMP-2 activity.

The senescent nature of adult mammalian cardiomyocytes is a major limiting factor that prevents regeneration resulting in heart failure. In this scenario, Alam et al. studied the significance of suppressing cell cycle inhibitors Rb1 and Meis2 to promote adult cardiomyocyte reentry to the cell cycle. Authors involved a gelatin and laponite^®^ nanocomposite hydrogel to deliver siRNA complexes and promote silencing of Rb1 and Meis2 following MI in rats [59]. Results from this study showed a significant increase in proliferation markers in adult cardiomyocytes with reduced infarct size and improved cardiac function post-MI.

In an interesting adaptation, a microextrusion-based transient transfection system was involved to transfer the GATA binding protein 4 (GATA4) plasmid to human umbilical cord-derived MSCs (hUC-MSCs) encapsulated in a thermoresponsive polyurethane (PU) hydrogel [87]. PU hydrogels induced GATA4-transfected hUC-MSCs to express the cardiac marker proteins and then differentiated into cardiomyocyte-like cells in 15 days. Moreover, resulting constructs led to in situ revival of heart function in zebrafish in 30 days.

In order to prevent siRNA nuclease-mediated hydrolysis when delivered systematically, an injectable, guest–host assembled hydrogel between PEI and PEG was developed [85]. siRNA polyplexes assembled with modified polymers improved transfection and viability of neonatal rat cardiomyocytes compared with PEI. When injected into rat myocardium, hydrogels localized polyplex release leading to GFP silencing for one week in a GFP-expressing rat.

MicroRNA-based therapies targeting cardiomyocytes have great potential for the treatment of MI. Certain miRNAs induce cardiomyocyte proliferation, sometimes leading to improve cardiac function [62,63,109]. Administration of these therapeutic miRNA via injectable hydrogels constitutes a potential approach to achieve a localized delivery into cardiac tissue [62,63]. Tian et al. observed that the miR-302/367 cluster plays an important role in cardiomyocytes proliferation during development inducing cardiomyocyte proliferation in the adult and promoting cardiac regeneration [109]. In view of their previous observations, these authors developed an injectable HA hydrogel for the local and sustained delivery of miR-302 mimics to the heart. A single injection of this hydrogel system in the mouse heart led to local and sustained cardiomyocyte proliferation for two weeks [62]. Likewise, a further decrease in cardiac end-diastolic and end-systolic volumes, and improved ejection fraction were observed four weeks after injection, compared with the controls.

### 2.3. Skin Tissue Repair

Chronic conditions such as diabetes mellitus or peripheral vascular disease can lead to impaired skin wound healing. Likewise, acute trauma such as degloving or large-scale thermal injuries are followed by a loss of skin organ function rendering the organism vulnerable to infections, thermal dysregulation, and fluid loss [110].

**Figure 5:**
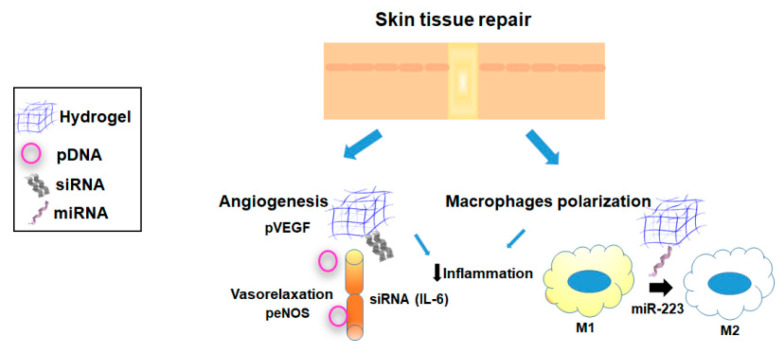
Main strategies involving the use of hydrogel-based nonviral gene delivery systems for skin tissue repair.

Oxygen plays a key role in wound healing, and hypoxia is a major cause of wound healing impairment. Consequently, treatments to improve hemodynamics and optimize wound oxygenation are compelling to stimulate the healing of these hypoxic tissues [111]. Delivery of therapeutic genes able to increase oxygenation in the wound tissue is an emerging tool to treat chronic wounds. Therefore, main strategies involving the use of hydrogels for gene delivery in wound healing are focused on increasing of angiogenesis or reducing the inflammation (Figure 5; Table 1 and Table 2).

Diabetic wound healing impairment is caused by a limited oxygen supply and a high oxygen consumption rate inside the wound. Moreover, diabetes wounds are characterized by an abnormal autoregulatory capacity from capillaries and a reduction in nitric oxide synthase (NOS) [112]. A decrease in NO production induces impaired vasorelaxation contributing to microvascular dysfunction [51,113]. In order to overcome this limitation, a collagen system based on collagen microspheres and a collagen hydrogel scaffold was involved to control the release of interleukin-6 (IL-6) siRNA and endothelial NOS (eNOS). The optimal doses of IL-6 siRNA and eNOS pDNA to decrease the volume fraction of inflammatory cells and increase the length density of blood vessels were confirmed at 7 and 14 days, respectively [45].

Angiogenesis is essential to wound healing. Thus, newly formed blood vessels participate in provisional granulation tissue formation and provide nutrition and oxygen to growing tissues [114]. Localized delivery of angiogenic factors such as VEGF via hydrogel systems offers a promising avenue to promote revascularization in wound healing [64,111,115]. A polycation jetPEI /VEGF-expressing plasmid complex was included in human fibrin sealant Crosseal to evaluate the relative efficiency of revascularization in a rat fasciocutaneous flap model [115]. Implantation of these systems resulted in increased flap survival at day 5 post-surgery compared with the controls receiving the matrix alone, due to an increased angiogenesis. Nevertheless, no differences were observed in flap survival between the group of rats receiving VEGF protein (the control group) and the animals receiving VEGF-expressing plasmid at this time point.

Macrophages play key roles in all phases of adult wound healing, namely, inflammation, proliferation, and remodeling [116]. In the course of normal wound healing process, classically-activated macrophages (M1) release proinflammatory cytokines during early stages of wound healing, while alternatively-activated macrophages (M2) finally resolve this inflammatory stage by secreting anti-inflammatory cytokines that promote wound healing. Non-healing chronic wounds, such as pressure, arterial, venous, and diabetic ulcers, indefinitely remain in the inflammation stage due to the persistence of M1 proinflammatory macrophages during the later stages of tissue repair [116]. Therefore, reprogramming of macrophages toward the M2 phenotype via miRNAs has emerged as a potential strategy for the treatment of these chronic disorders. Saleh et al. developed adhesive hydrogels containing miR-223 5p mimic (miR-223)-loaded HA nanoparticles to control tissue macrophages polarization during wound healing processes [60]. These adhesive systems could adhere to and cover the wounds during the healing process in an acute excisional wound model. Further, local delivery of miR-223 efficiently prompted the formation of uniform vascularized skin at the wound site, due to the polarization of macrophages to the M2 phenotype.

### 2.4. Nervous Tissue Repair

**Figure 6:**
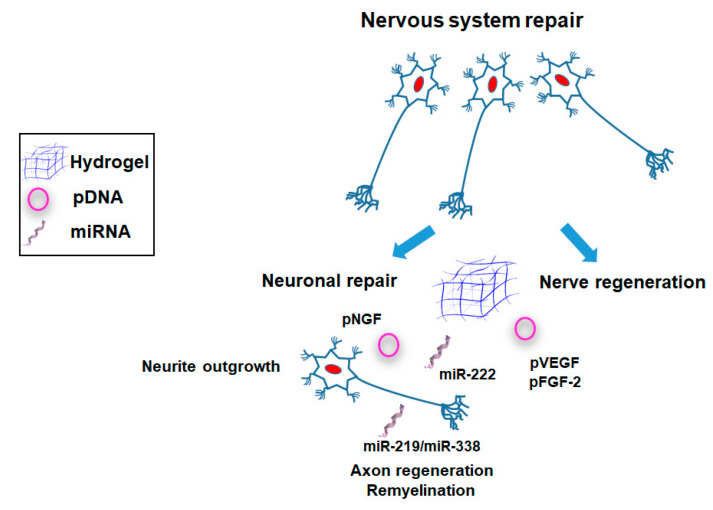
Main strategies involving the use of hydrogel-based gene delivery systems for nervous system repair.

Gene therapy constitutes a potential tool to treat central nervous system (CNS) disorders. However, its use is still limited by important hurdles as the intrinsic difficulty of treating neurological disorders [117,118]. Brain is a complex organ in which disease processes induce a broad spectrum of pathological states affecting the neural development, plasticity, and metabolism. Herein, the nature of pathophysiology of neurodegenerative diseases is often not completely understood, limiting the development of new treatments. Alongside, brain access is limited by the blood–brain barrier (BBB) that prevents the delivery of therapeutic agents to the CNS and particularly the use of systemic treatments [118]. The versatility of gene delivery and the mechanics and tailorability of hydrogels makes gene delivery from hydrogels an attractive approach for nervous tissue regeneration. These systems can provide a combinatorial approach for nerve regeneration, with the hydrogel supporting neurite outgrowth and gene delivery inducing the expression of inductive factors (Figure 6; Table 1 and Table 2).

Jaclyn et al. modified PEG hydrogels with affinity peptides (K4, K8) to increase pDNA encoding for nerve growth factor (NGF) lipoplexes retention [72]. Transfection was increased 5- to 15-fold with K8 and K4, respectively, over the Arg-Gly-Asp (RGD) control peptide. Interestingly, while vector retention was similar in K8- and K4-modified hydrogels, vector dissociation rate was reduced for K8, due to a more excessive binding. When tested in an in vitro co-culture model, K4-modified hydrogels promoted maximal neurite outgrowth. More recently, same authors gelled enzymatically-degradable PEG hydrogels encapsulating dorsal root ganglia explants, fibroblasts, and lipoplexes encoding nerve growth factor to physically guide neurite outgrowth [55]. Transfection of fibroblasts was enhanced with increasing concentration of RGD cell adhesion sites and decreasing PEG content from hydrogels. In addition, neurite length raising was maximal within 7.5% PEG hydrogels at intermediate RGD levels and increased with lipoplexes delivery.

In another approach, a nanostructured conduit made of biocompatible and biodegradable poly(ε-caprolactone) (PCL) and filled with fibrin hydrogel matrix in combination with local delivery of expression plasmids carrying genes encoding VEGF and FGF-2 was implanted in a rat sciatic nerve with a nerve diastasis [53]. Direct local injection of plasmid to the site of traumatic injury stimulated regeneration of rat sciatic nerve and recovery of motor and sensory functions.

Spinal cord injuries (SCI) often lead to persistent neurological dysfunction due to failure in axon regeneration. Design of 3D aligned nanofiber–hydrogel scaffolds as biofunctionalized platforms to provide contact guidance and sustained gene delivery, constitutes an attractive approach for nerve injury treatment. Nguyen and coworkers synthetized an aligned poly(ε-caprolactone-co-ethyl ethylene phosphate) (PCLEEP) electrospun scaffold distributed in a 3D collagen hydrogel for in vivo delivery of neurotrophin-3 (NT-3) as the model protein and miR-222 as the model microRNA [46]. Among selected factors, NT-3 is known to promote neuronal survival, axonal sprouting, and regeneration [119]. In addition, miR-222 is enriched in axons and participates in controlling local protein synthesis at distal axons [120]. When tested in a hemi-incision model at cervical level 5 from rat spinal cord, constructs led to aligned axon regeneration at one-week post-injury. More recently, the same authors involved the same hydrogel scaffolds for improving differentiation, maturation, and myelination of oligodendrocytes (OL) [47]. To this end, they incorporated miR-219/miR-338 into the scaffolds to enhance remyelination after a hemi-incision injury at C5 level of Sprague–Dawley rats. These miRNA are known to promote OL progenitor cells (OPSCs) differentiation in vitro and in vivo by suppressing the expression of gene targets that promote OPC proliferation [121]. Results showed that rats implanted with miR-219/miR-338-loaded scaffolds retained a higher population of oligodendroglial lineage cells around the lesion site.

## 3. Discussion

Nonviral gene delivery via hydrogels systems has emerged as a potential approach in various regenerative medicine scenarios in order to activate tissue reparative processes. The design of these systems requires the identification of the most adapted nonviral carriers to complex NA molecules (lipids, polycations, micelles, etc.), biomaterial class (nature, properties, route of administration), and NA molecules (growth factors, transcription factors, anti-inflammatory molecules, signaling agents, etc.) in order to promote an effective transgene expression.

Due to its biosafety, current worldwide approved nonviral gene products have been mainly focused on the administration of naked pDNA molecules encoding for angiogenic factors (pDNA-HGF: Collategene [122]; pDNA-VEGF: Neovasculgen [123]) for the treatment of cardiovascular diseases [124]. Of note, the first RNAi drug product (Onpattro*)* was approved in 2018 and is based on a nonviral lipid nanoparticle and siRNA [125] for the treatment of hereditary transthyretin amyloidosis.

Yet, even though numerous studies conducted in clinically relevant animal models in vivo reinforce the potential of hydrogel-based nonviral gene delivery for the treatment of different pathologies [35,50,56,57,58,59,60,92], there are not, to the best of our knowledge, clinical trials thus far reporting the feasibility of exploiting these adapted biomaterials to deliver nonviral vectors.

Most of the efforts on musculoskeletal tissue repair have been concentrated on bone and cartilage tissues via delivery of pDNA encoding for osteogenic (BMP-2, OSX) or chondrogenic (TGF-β3, SOX9) factors [33,35,36,37,38,40,41,42,70,83,90], osteogenic siRNA [44,49,71,78,81], mechanosensitive osteogenic/angiogenic miRNA [78,80,82,84], and chondrogenic mRNA molecules [17] using alginate [33,34,35,36,37,38], fibrin [17,49], chitosan [41,42,44], gelatin [82,90], or PEG-based hydrogels [70,71,78,80,81,83,84].

Hydrogel-based gene delivery for cardiovascular tissue repair has been focused on the delivery of pDNA encoding for angiogenic factors (VEGF) for inducing neovessel growth and prevent stent thrombosis [39,50,52,57,61] via fibrin- [50,52], HA- [52,61], alginate- [39], or gelatin-based hydrogels [57]. Other strategies have been centered on improving cardiomyocytes function by delivering RNA molecules to prevent their senescence (siRNA) [59], or inducing their proliferation (miRNA) [62,63] via gelatin [59] or HA hydrogels [62,63].

The main strategies in gene delivery to promote wound healing have been directed to promote the angiogenesis or reduce the inflammation. Herein, different hydrogels based on natural polymers such as fibrin [115], HA [64], or gelatin [60] have been produced for delivering pDNA (VEGF) [64,115] or miRNA to promote macrophages reprogramming [60].

Finally, gene delivery for nervous tissue repair has been mainly focused on promoting neuronal and/or nerve repair. Delivery of pDNA encoding for various angiogenic growth factors (NGF, VEGF, and FGF-2) [53,72] or miRNA [46,47] to promote axon remyelination have been described using PEG- [72], fibrin- [53], or collagen-based [46,47] hydrogels.

In conclusion, controlled delivery of nonviral gene transfer vectors from hydrogels represents a promising, growing area of research for the future effective and safe treatment of a number of human pathologies. This strategy may help to circumvent current limitations from nonviral gene therapy and provide tunable platforms adapted to the tissue to be repaired.

## Figures and Tables

**Table 1:** Controlled nonviral gene delivery from natural-based hydrogels.

Polymer	System	NA Type	Study	Application	Ref.
Alginate	Alginate hydrogel	pDNA encoding for BMP-2 (calcium phosphate NPs)	In vitro (MC3T3-E1 cells)/In vivo (s.c. injection in mice)	Bone repair	[33]
	Alginate hydrogel	pDNA encoding for BMP-2	In vitro (MSCs)/In vivo (s.c. dorsal pocket from nude mice)	Bone repair	[34]
	Alginate hydrogel	pDNA encoding for BMP-2 (His polyplexes)	In vivo (i.m. implantation in goats)	Bone repair	[35]
	Alginate hydrogel	pDNA encoding for BMP-2 (acetylated PEI and cationic polysaccharide complexes)	In vitro (gMSCs)/In vivo (s.c. dorsal pocket from mice)	Bone repair	[36]
	Alginate hydrogel	pDNA encoding for BMP-2 or TGF-β3 (nHA complexes)	In vitro (MSCs)	Osteochondral repair	[37]
	Alginate/methyl-cellulose hydrogel	pDNA encoding for BMP-2, TGF-β3 or SOX9 (RALA and nHA complexes)	In vitro (MSCs)/In vivo (s.c. implantation in mice back)	Osteochondral repair	[38]
	Calcium alginate hydrogels	pDNA encoding for VEGF (PEI polyplexes)	In vitro (MC3T3-E1 cells)/In vivo (injection in mice)	Therapeutic angiogenesis	[39]
Cellulose	CMC/bPEI nanogels	pDNA encoding for OSX-GFP (bPEI-modified CMC nanogels)	In vitro (MSCs)	Bone repair	[40]
Chitosan	Chitosan-based hydrogel with α,β-GP	pDNA encoding for BMP-2 (chitosan NPs)	In vitro (human periodontal ligament cells)	Bone repair	[41]
	Chitosan hydrogel	pDNA encoding for BMP-2 (chitosan NPs)	In vivo (i.m. injection in rats)	Bone repair	[42]
	Chitosan hydrogel	siRNA against murine RANK	In vivo (sc injection in mice)	Periodontal tissue repair	[43]
	Methacrylated glycol chitosan hydrogel	siRNA against Noggin (cationic steorosomes)	In vitro (hMSCs)/In vivo (mouse calvarial defects model)	Bone repair	[44]
Collagen	Collagen microspheres within collagen hydrogel	pDNA encoding for eNOS/siRNA against IL-6 (dPAMAM polyplexes)	In vivo (s.c. implantation in rats)	Therapeutic angiogenesis	[45]
	PCLEEP nanofibers-collagen hydrogel	miRNA-222 (PCL-PPEEA micellar NPs)	In vivo (rat spinal cord incision model)	Nerve repair	[46]
	Aligned electrospun fibers-collagen hydrogel	miRNA-219 and miRNA-338 (TransIT-TKO complexes)	In vitro (rat oligodendrocytes)/In vivo (rat spinal cord incision model)	Nerve repair	[47]
Dextran	Succinate–modified dextran hydrogel	siRNA against GFP (LPF lipoplexes)	In vitro (HeLa cells)	n.s.	[48]
Fibrin	Fibrin hydrogel	siRNA against Noggin (LPF complexes)	In vitro (MC3T3-E1 cells)	Bone repair	[49]
	Fibrin hydrogel	mRNAs encoding SOX9 or MYOD (3DfectIN^®^ complexes)	In vitro (hMSCs)	Muscle and cartilage repair	[17]
	Fibrin hydrogel	pDNA encoding for VEGF and Angiopoietin 1 (Tat peptide NPs or NPs hybridized to PAA wrapped single-walled carbon nanotubes)	In vivo (balloon-injured canine femoral artery model from dogs)	Cardiovascular tissue repair	[50]
	Fibrin microspheres	pDNA encoding for eNOS (fibrin complexes)	In vivo (rabbit ear ulcer model)	Wound healing	[51]
	Fibrin hydrogel or HA hydrogel	pDNA encoding for VEGF or β-gal (PEI polyplexes)	In situ (CAM)	Wound healing	[52]
	PCL matrix filled with fibrin hydrogel	pDNA encoding VEGF-and FGF-2	In vivo (implantation in rats)	Nerve repair	[53]
	Poloxamine/fibrin hybrid hydrogels	pDNA encoding for GFP (jetPEI polyplexes)	In vitro (N2A cells)	Soft tissue repair	[54]
	PEG, fibrin or HA hydrogel	pDNA (PEI polyplexes or LPF lipoplexes)	In vitro (NIH/3T3)/In situ (CAM)	n.s.	[55]
Pullulan	Cationized pullulan hydrogel	siRNA against MMP- 2 (DEAE-pullulan complexes)	In vivo (implantation in rabbits)	Cardiovascular tissue repair	[56]
Gelatin	Gelatin hydrogel	pDNA encoding for VEGF (PEI-GO nanocomplexes)	In vitro (cardiomyocytes)/In vivo (rat myocardial infarction model)	Cardiovascular tissue repair	[57]
	Polyester stent grafts coated with cationized gelatin hydrogel	mRNA encoding *lac*Z	In vivo (implantation in aortic wall of rabbits)	Cardiovascular tissue repair	[58]
	Gelatin/silicate NPs composite hydrogel	siRNA against Rb1 and siRNA against Meis2 (LPF lipoplexes)	In vitro (human cardiomyocytes)/In vivo (rat myocardial infarction model)	Cardiovascular tissue repair	[59]
	Gelatin methacryloyl hydrogel	miRNA-223 (HA NPs)	In vitro (macrophages)/In vivo (mice full-thickness wound model)	Wound healing	[60]
HA	Thiol modified HA/PEG-DA hydrogel	miRNA-COX1 and miRNA-COX2 (PEI-PLGA NPs)	In vitro (tenocytes)/In vivo (chicken tendon injury model)	Flexor tendon repair	[92]
	HA hydrogels	pDNA encoding for VEGF or GFP (PEI polyplexes)	In vivo (implantation in mice)	Therapeutic angiogenesis	[61]
	HA hydrogel	miRNA-302	In vitro (mouse cardiomyocytes)/In vivo (injected in non-infarcted hearts of mice)	Cardiovascular tissue repair	[62]
	Elastin-like protein-HA hydrogel	miRNA-199a-3p (PEG NPs)	In vitro (hESC-CMs and hESC-ECs)/In vivo (rat myocardial infarction model)	Cardiovascular tissue repair	[63]
	HA hydrogel modified with MMPs	pDNA encoding for VEGF or GFP-*Luc* (PEI polyplexes)	In vivo (mouse wound healing model)	Wound healing	[64]
	HA hydrogel functionalized with Norb	pDNA encoding for G*Luc* (jetPEI polyplexes)	In vitro (human dermal fibroblasts)	Wound healing	[65]
	HA hydrogel functionalized with MMPs	pDNA encoding for G*Luc* (PEI polyplexes)	In vitro (MSCs)	n.s.	[69]
	HA hydrogel	pDNA encoding for G*Luc* (cationic nioplexes)	In vitro (MSCs)	n.s.	[66]
	Microporous HA hydrogel	pDNA encoding for G*Luc* (PEI-polyplexes)	In vivo (implantation in mice)	n.s.	[67]
	HA hydrogel functionalized with MMPs	pDNA encoding for G*Luc* or SEAP (PEI polyplexes)	In vitro (HEK293T)	n.s.	[68]

Abbreviations: pDNA: plasmid DNA; BMP-2: bone morphogenetic protein 2; NPs: nanoparticles; MC3T3-E1: osteoblast cell line; MSCs: mesenquimal stem cells; s.c.: subcutaneous; His: histidine; i.m.: intramuscular; PEI: polyethylenimine; gMSCs: goat mesenchymal stem cells; TGF-β3: transforming growth factor β3; SOX-9: sex-determining region Y-type high mobility group box 9; RALA: arginine-alanine-leucine-arginine amphipathic peptide; nHA: nanohydroxyapatite; VEGF: vascular endothelial growth factor; OSX-GFP: osterix-green fluorescent protein; bPEI: branched poly(ethyleneimine); α,β-GP: α,β-glycerophosphate; CMC: carboxymethylcellulose; siRNA: small interfering RNA; RANK: receptor activator of nuclear factor-Κb; eNOS: endothelial nitric oxide synthase; IL-6: interleukin-6; dPAMAM: polyamidoamine dendrimer; s.c.: subcutaneous; PCLEEP: poly (ε-caprolactone-co-ethyl ethylene phosphate); miRNA: microRNA; PCL:-PPEEA: poly(ε-caprolactone)-block-poly(2-aminoethyl ethylene phosphate); NPs: nanoparticles; GFP: green fluorescent protein; LPF: lipofectamine; MC3T3-E1: osteoblast cell line; mRNA: messenger RNA; MYOD: myoblast determination protein 1; PAA: polyacrylic acid; β-gal: β-galactosidase; CAM: chorioallantoic membrane; PCL: poly (ε-caprolactone); FGF-2: fibroblastic growth factor 2; N2A: mouse neuroblastoma cell line; HA: hyaluronic acid; PEG: polyethylene glycol; NIH/3T3: mouse fibroblasts cell line; n.s.: not specified; MMP-2: metalloproteinase 2; DEAE: diethylaminoethylamine; GO: graphene oxide; *lacZ*: β-galactosidase gene; Rb1: retinoblastoma gene; Meis2: homeobox protein Meis2; PEG-DA: polyethylene glicol dyacrylate; COX: cyclooxygenase; PLGA: poly(lactic-co-glycolic acid); hESC-CMs: human embryonic stem cell-derived cardiomyocytes; hESC-ECs: human embryonic stem cell-derived endothelial cells; n.s.: not specified; GFP-*Luc*: green fluorescent protein-luciferase; Norb: norbornene; G*Luc:* gaussian luciferase; SEAP: secreted embryonic alkaline phosphatase; HEK293T: human embryonic kidney 293 cells.

**Table 2:** Controlled nonviral gene delivery from synthetic-based hydrogels.

Polymer	System	NA Type	Study	Application	Ref.
PEG	PEG hydrogel membrane	pDNA encoding for BMP-2 (lipoplexes)	In vitro (hFOB cells)/In vivo (calvarial model pig)	Bone repair	[70]
	PLA-DX-PEG hydrogel	siRNA against Noggin	In vivo (implantation in dorsal muscle pouches from mice)	Bone repair	[71]
	PEG hydrogel	siRNA against GFP (PEI polyplexes)	In vitro (hMSCs)	Bone repair	[75]
	PEG-DA or PEG- DPA hydrogel	siRNA against Noggin or miRNA-20a (PEI polyplexes)	In vitro (hMSCs)	Bone repair	[78]
	PEG/PLA/DM hydrogel	siRNA against WW domain-containing E3 ubiquitin protein ligase 1 (polymeric NPs)	In vitro (MSCs)/In vivo (murine femoral fracture model)	Bone repair	[79]
	HP/HA/PEG composite hydrogel	miRNA-26a (siPORT NeoFX complexes)	In vitro (mBMMSCs and hBMMSCs)/In vivo (mouse critical size calvarial bone defect model)	Bone repair	[80]
	PEG hydrogel	miRNA-20a (PEI polyplexes)	In vitro (hMSCs)/In vivo (rat calvarial bone defect model)	Bone repair	[81]
	Gelatin/PEG hydrogel	miRNA-100-5p and miRNA-143-3P (PEI complexes)	In vitro (MSCs)	Bone repair	[82]
	OPF porous scaffold	pDNA encoding for BMP-2 or SOX trio (PEI-nHA complexes)	In vivo (rat knee osteochondral defect model)	Osteochondral repair	[83]
	MMP-responsive PEG/peptide hydrogel	miRNA-29 (PGPC polyplex micelles)	In vitro (nucleus pulposus cells)/In vivo (rat intervertebral disc degeneration model)	Fibrocartilage repair	[84]
	PEI/PEG hydrogel	siRNA against GFP (PEI polyplexes)	In vivo (injection into myocardium of rats)	Cardiovascular tissue repair	[85]
	PEG-vinyl sulfone hydrogel modified with cysteine residues	pDNA encoding for EGFP-*Luc* or NGF (TransFast lipoplexes)	In vitro (dorsal root ganglia explants from chicken embryos)	Nerve repair	[72]
	PEG hydrogel	pDNA encoding for GFP-*Luc* or NGF (TransFast lipoplexes)	In vitro (HT-1080 cells or primary neuron clusters from chicken eggs)	Nerve repair	[77]
	PEG-vinyl sulfone hydrogel	pDNA encoding for *Luc* (Cationic bolaamphiphile complexes)	In vitro (MSCs)	n.s.	[73]
	PEG-gelatin hydrogel	pDNA encoding for *Luc* or GFP (PBAEs and PAAs polyplexes)	In vitro (HEK293T cells)	n.s.	[74]
	PEG/DTT hydrogel	siRNA against mTOR	In vitro (3T3 fibroblasts)/In vivo (s.c. implantation in mice)	n.s.	[76]
PNIPAm	PNIPAm/LDH hydrogel	siRNA against GAPDH (LPF lipoplexes)	In vivo (s.c. injection in mice)	Cartilage repair	[86]
Polyurethane	Polyurethane hydrogel	pDNA encoding for GATA4 (naked, microextrusion-based transfection system)	In vitro (hUC-MSCs)	Cardiovasculartissue repair	[87]
Poly (organophosphazene)	Poly(organophosphazene) thermosensitive hydrogel	pDNA (GC-g-PEI complexes)	In vitro (HepG2 cells)/In vivo (injection in mice)	Hepatocyte targeting	[88]

Abbreviations: PEG: polyethylene glycol; pDNA: plasmid DNA; BMP-2: bone morphogenic protein 2; nFOB: human fetal osteoblastic cell line; PLA-DX-PEG: poly-D,L-lactic acid-p-dioxanone-polyethylene glycol block co-polymer; siRNA: small interfering RNA; GFP: green fluorescent protein; PEI: polyethyleneimine; hMSCs: human mesenquimal stem cells; PEG-DA: poly(ethylene glycol)-diacrylate; PEG-DPA: poly(ethylene glycol)-diphotodegradable-acrylate; miRNA: microRNA; PEG/PLA/DM: poly(ethylene glycol)-b-poly(lactide)-b-dimethacrylate; NPs: nanoparticles; HP/HA/PEG: thiol-modified analog of heparin with thiol-modified hyaluronan and poly(ethylene glycol) diacrylate; mBMMSCs: murine bone marrow mesenquimal stem cells; hBMMSCs: human bone marrow mesenquimal stem cells; OPF: oligo[poly(ethylene glycol) fumarate]; SOX trio: sex-determining region Y-type high mobility group box 5,6 and 9; nHA: nanohydroxyapatite; MMP: metalloproteinase; PGPC: poly(ethylene glycol)-GPLGVRG-poly{*N*′-[*N*-(2-aminoethyl)-2-aminoehtyl]aspartamide}-cholesteryl(PEG-GPLGVRG-PAsp (DET)-Chole); EGFP-*Luc*: firefly luciferase/enhanced green fluorescent fusion protein; NGF: nerve growth factor; HT-1080: fibrosarcoma cell line; n.s.: not specified; HEK-293T: human embryonic kidney 293 cells; PBAEs: poly(β-amino)esters; PAAs: poly(amido amine)s; DTT: dithiothreitol; mTOR: mammalian target of rapamycin; PNIPAm: poly(*N*-isopropylacrylamide); LDH: layered double hydroxides; GAPDH: glyceraldehyde-3-phosphate dehydrogenase; LPF: lipofectamine; s.c.: subcutaneous; GATA4: transcription factor; hUC-MSCs: human umbilical cord-derived mesenchymal stem cells; GC-g-PEI: galactosylated chitosan-graft-polyethylenimine; HepG2: human liver cancer cell line.
